# Evaluation of a topical formulation of eprinomectin against *Anopheles arabiensis* when administered to Zebu cattle (*Bos indicus*) under field conditions

**DOI:** 10.1186/s12936-016-1361-z

**Published:** 2016-06-17

**Authors:** Saul Lozano-Fuentes, Rebekah C. Kading, Daniel A. Hartman, Edward Okoth, Naftaly Githaka, Vishvanath Nene, Richard M. Poché

**Affiliations:** Genesis Laboratories, 10122 NE Frontage Rd, Wellington, CO USA; Department of Microbiology, Immunology and Pathology, Colorado State University, Fort Collins, CO USA; International Livestock Research Institute, P.O. Box 30709, Nairobi, Kenya

**Keywords:** *Anopheles arabiensis*, Eprinomectin, Endectocide, Malaria, Cattle, Kenya

## Abstract

**Background:**

Although vector control strategies, such as insecticide-treated bed nets (ITNs) and indoor residual spraying (IRS) have been effective in Kenya the transmission of malaria continues to afflict western Kenya. This residual transmission is driven in part by *Anopheles arabiensis*, known for its opportunistic blood feeding behaviour and propensity to feed outdoors. The objective of this research was to evaluate the efficacy of the drug eprinomectin at reducing malaria vector density when applied to cattle (*Bos indicus*), the primary source of blood for *An. arabiensis*, under field conditions.

**Methods:**

A pilot study was carried out in the Samia District of western Kenya from September to October of 2014. Treatment and control areas were randomly designated and comprised of 50 homes per study area. Before cattle treatments, baseline mosquito counts were performed after pyrethrum spray. Cows in the treatment area were administered topical applications of eprinomectin at 0.5 mg/kg once a week for two consecutive weeks. Mosquito collections were performed once each week for two weeks following the eprinomectin treatments. Mosquitoes were first identified morphologically and with molecular confirmation, then screened for sporozoite presence and host blood using PCR-based methods.

**Results:**

The indoor resting density of *An. arabiensis* was significantly reduced by 38 % in the treatment area compared to the control area at one-week post-treatment (Control mean females per hut = 1.33 95 % CI [1.08, 1.64]; Treatment = 0.79 [0.56, 1.07]). An increase in the indoor resting density of *Anopheles gambiae* s.s. and *Anopheles funestus* s.s. was observed in the treatment area in the absence of *An. arabiensis.* At two weeks post-treatment, the total number of mosquitoes for any species per hut was not significantly different between the treatment and control areas. No change was observed in *An. arabiensis* host preference as a result of treatment.

**Conclusions:**

Systemic drugs may be an important tool by which to supplement existing vector control interventions by significantly impacting outdoor malaria transmission driven by *An. arabiensis* through the treatment of cattle.

## Background

Kenya experiences an estimated 6.7 million new clinical cases of malaria, with approximately 4000 deaths each year [1]. As a result, it is the leading cause of morbidity in children in western Kenya [2]. The three predominant malaria vectors in western Kenya, *Anopheles gambiae* sensu stricto (s.s.), *Anopheles arabiensis*, and *Anopheles funestus* s.s., have undergone changes in their relative abundance over the last 10 years, most likely in response to the pressures of traditional control strategies, such as indoor residual spraying (IRS) and insecticide-treated nets (ITN) [3, 4]. These control methods have been implemented intensively throughout many areas of Kenya and other parts of Africa and have led to a reduction in the abundance of indoor biting (endophagic) and indoor resting (endophilic) vector populations in some areas [3, 4]. Even with the increasing coverage of ITNs in western Kenya, there has been a resurgence of malaria transmission suggesting that this traditional vector control method may no longer be as effective in reducing malaria transmission in this region [5].

Complicating the efficacy of ITNs and IRS is the tendency for some mosquitoes to feed outdoors and on alternative hosts. In particular, *An. arabiensis* is known to feed preferentially on cattle and rest outside of human habitations [6–8], where it is unlikely to encounter control strategies which target endophilic mosquitoes. In the area west of Kisumu, Kenya, *An. arabiensis* fed most frequently on cattle (65 % of blood meals; 22 % mixed bovine/human; 13 % human) while *An. gambiae* s.s. fed mostly on humans (70 % of blood meals; 21 % mixed human/bovine; 9 % bovine) [4]. In areas where *An. arabiensis* was observed to be more anthropophagic, they still predominantly fed outdoors where ITN and IRS strategies are not effective [9]. Mwangangi et al. [10] reported that in the Taveta district, along the coast of Kenya, *An. arabiensis* and *Anopheles coustani* were responsible for the highest number of infectious bites per person per year. In another study, the percentage of the annual entomological inoculation rate (EIR) contributed by *An. arabiensis,* in 30 sites along the Kenyan coast, ranged from 0 to 64 % [11]. Therefore, there is the need to develop a novel vector control strategy that targets outdoor biting malaria vectors which do not encounter IRS and ITNs.

The current options for targeting outdoor-biting vectors in urban areas consists of insecticidal fogs or ultra-low volume (ULV) sprays. Neither of these interventions is practical or useful in rural settlements, where much of the malaria transmission in Africa occurs and are prohibitively expensive for large-scale application. Insecticidal fogging and ULV are also risky for use in agricultural areas due to their secondary effects on agriculturally beneficial insects, such as bees [12]. In this study, cattle was treated, the primary blood meal source for some species and populations of malaria vectors, with the active ingredient (AI) eprinomectin. Systemic circulation of eprinomectin in the blood reduces mosquito survival after the acquisition of a blood meal [13].

Treatment of cattle with a systemic insecticide has already been explored for the control of the *Leishmania* spp. vectors (*Phlebotomus argentipes* and *Phlebotomus papatasi*) in India and Tunisia [14–16], and of *Anopheles* mosquito populations in Kenya [17]. Fritz et al. [17] found that the survivorship and fecundity of *An. gambiae* s.s. was significantly reduced after mosquitoes fed on cattle treated with ivermectin [17]. Specifically, 90 % of the *An. gambiae* s.s. that fed on the ivermectin-treated cattle within 2 weeks of treatment did not survive longer than 10 days post-blood meal, and no eggs were produced by *An. gambiae* s.s. that fed on ivermectin-treated cattle within 10 days of treatment [17]. Alout et al. [18] also reported a significant effect on mosquito age structure (parity) for 3 weeks following a mass drug administration of ivermectin to people. By impacting both mosquito survivorship and fecundity, this treatment strategy effectively reduces mosquito vectorial capacity for transmission of malaria parasites through two simultaneous mechanisms. The vector population density is reduced, and the probability of the vector to survive through the extrinsic incubation period is also decreased. By capitalizing on the propensity of *An. arabiensis* to feed on cattle in addition to people, it was hypothesized that treatment with systemic insecticides of cattle will ultimately reduce residual malaria transmission to humans by *An. arabiensis* through a combined reduction in mosquito population density and survivorship.

Our study targeted adult, host-seeking *An. arabiensis* via treatment of their preferred host: cattle. There were two specific aims of this study. The first was to determine the efficacy of topical eprinomectin treatments at reducing the population density of *An. arabiensis* under field conditions. The second was to examine the effect of the treatment on additional entomological parameters such as sporozoite rates and mosquito blood feeding patterns.

## Methods

### Study area

This study took place between Busia (Longitude 34.11101°, Latitude 0.45822°), and Sio Port (Longitude 34.02222°, Latitude 0.21875°), in western Kenya along the coast of Lake Victoria at an approximate altitude of 1200 meters above mean sea level. This region of Kenya is classified as a tropical wet-dry climate with average temperatures ranging between 19 and 29 °C, and precipitation averaging 1200 mm annually [19].

Eprinomectin treatment of cattle was randomly designated to one of two sites, each site consisting of 50 huts (for a total of 100 huts) in which villagers sleep. The treatment and control sites were separated by a distance of approximately 1 km. A buffer zone of 0.5 km surrounded the treatment site; cattle located in it were treated. Both sites were located at least 0.5 km away from extensive swamps or lake habitats.

Verbal and written consent was obtained from the head of each household participating in this study. The consenting participants signed a corresponding consent form for whether their home was located in a treatment area (mosquito collections and cattle treatments), control area (mosquito collections only) or the surrounding buffer area (cattle treatments only). Consent forms were translated into common local languages: Kiswahili and Samia. Homesteads that declined involvement at the moment of consent or at any time during the study were not included.

The local health district officer and village chiefs reported that no malaria control activities were carried by NGOs or governmental agencies during the study. Nevertheless, bed nets were commonly observed in participants’ huts.

### Cattle treatment

Zebu cattle (*Bos indicus* local breed, aged over 6 months) in the treatment and buffer area, from participating household, received two treatments of eprinomectin (Eprinex^®^ POUR-ON. Each of mL contained 5 mg of eprinomectin, CAS 123997-26-2, MERIAL Ltd.) at a dose of ~1 mL/10 kg body weight (0.5 mg eprinomectin per kilogram of body weight). Dosing occurred once per week during the two consecutive weeks following the baseline mosquito collections. Cattle in the control group received no treatment before or after the study. A veterinarian was present during the application of the active ingredient and was on call in the event of any adverse effects in the cattle. Treatment of animals was approved by the ILRI Animal Care and Use Committee, IACUC-RC2015-08.

### Entomological impact

The mosquito collections were conducted between September and October 2014. Both indoor and outdoor mosquito resting populations were surveyed before the cattle treatments to develop a baseline. Indoor populations were assessed using pyrethrum spray catches (PSC) [20], while outdoor populations were evaluated by placing two clay pots outside each hut, for a total of 400, in the treatment and control areas following Odiere et al. [21]. However, bags fashioned from a netting material with a drawstring were placed over the pot openings in place of aspirating the resting mosquitoes. The bags were extended upwards while the pots were shaken to help the escaping mosquitoes to enter the bags, this was followed by a visual inspection of the interior. If insects were still present inside a second bag was used and a second pot shake was performed. The clay containers were positioned for at least 2 days, and up to 1 week, for acclimation before the mosquito collections. On collection days, indoor and outdoor mosquitoes were collected between the hours of 0700 and 1100.

Post-treatment indoor and outdoor collections were conducted at week one and week two after the second cattle treatment. Each mosquito was transferred from field collections into 0.6 mL tubes containing silica gel desiccant for the preservation of DNA and transport to Genesis Laboratories (Wellington, CO, USA) for species identification and molecular analysis [22].

### Mosquito identification, blood meal source, and sporozoite rates

*Anopheles* mosquitoes were first sorted by sex and then morphologically identified to species complex, including *An. gambiae* sensu lato (s.l.) or *An. funestus* s.l. [23, 24]. Male mosquitoes were excluded from the study while females were further identified to species level via molecular analysis [25, 26].

DNA was extracted separately from abdomens and head/thoraces [27]. A multiplex polymerase chain reaction (PCR) was employed to detect the presence of single or mixed blood meals from humans, cattle, dogs, pigs, and goats from mosquito abdomen DNA [27]. Head/thorax DNA extractions were screened for *Plasmodium falciparum* and *Plasmodium ovale* DNA by nested PCR [28], with the assumption that *P. falciparum* DNA detected in the head/thorax of the mosquito represented the presence of sporozoites. The mosquito sporozoite infection rate was calculated as the percentage of *Plasmodium* positive specimens divided by the total number of samples from each species and site.

### Data analysis of entomological impact parameters

Bayesian inference was selected in place of Null Hypothesis Significance Testing (NHST) to make full use of the data structure and avoid data transformation. Estimations can be compared without having to make approximations or assumptions typically made in NHST (e.g., homogeneity of variances across groups, normally distributed noise, normalizing data, etc.).

### Blood meal proportions

Blood meal proportions (*p*) were estimated using Bayesian inference. For each of blood meal hosts (*k*), the number of blood meals was assumed to follow a multinomial distribution with an unknown parameter *p*,

1 $$y\left[ {1 \ldots k} \right]\;\sim \;Multinomial\;\left( {p\left[ {1 \ldots k} \right],\;N} \right).$$

Bayesian inference uses prior knowledge to modify the likelihood function Eq. (1). The lack of data about blood host preferences in the area was described mathematically using non-informative priors and giving the same weight to each host, dividing the shape parameter *α* by the total number of classes.

2 $$p\left[ {1 \ldots k} \right]\;\sim \;Dirichlet\left( {\alpha \left[ {1 \ldots k} \right] = \frac{1}{k} } \right).$$

The most probable value of the unknown parameter was searched using Markov Chain Monte Carlo (MCMC) simulations with four chains, a burn-in (discarded iterations) of 10,000 and 100,000 iterations. JAGS 4.0 for Linux and the rjags 4.6 libraries [29, 30] for the R language [31] was used to run the simulations; chain convergence and autocorrelation were assessed using the CODA R package [32]. For the estimated parameters 95 % credibility intervals [33] were obtained.

### Sporozoite rate

For determining the sporozoite rate (*r*) of each malaria vector species, it was assumed that positive and negative females would follow a Binomial distribution (*y* ~ Binomial (*r*, n)). Since no previous knowledge of the sporozoite rate in the area was available, a non-informative prior was used drawn from a flat Beta distribution (*r* ~ Beta (1, 1)) giving equal weight to all possible values of *r*. The same software and procedures as in the blood meal multinomial evaluation were used.

### Mosquito density

To estimate the female indoor resting density (µ) of *An. gambiae* s.s., *An. arabiensis,* and *An. funestus* s.s., it was assumed that the number of females per hut followed a Poisson distribution with mean µ. The Poisson distribution uses only one parameter to describe both the mean and the standard deviation. The lack of previous information during the baseline collection was expressed with a non-informative prior [µ ~ Log-normal(mean = 0, standard deviation = 100,000)]. However, to reduce the estimation’s uncertainty a strategy in which the results from the previous analysis served as priors for the subsequent one was followed (i.e., the mean and standard deviation of µ obtained from the baseline data served as priors for the first-week analysis, and so on).

## Results

### Cattle treatment

In the treatment area, 79 adults and five calves in were treated. In the buffer area that surrounded the treatment households, 503 adult cows and 31 calves were also treated. While no cattle were processed in the control area, a total of 75 adult cattle and five calves was reported by the participants.

Cattle body weights (BW) were estimated visually using local expert opinion before treatment. Given the significant variation in cattle rearing practice among households, local veterinarians judged the use of BW estimation by girth measurements to be inadequate. At the time of dosing, the landscape did not allow cattle movement to a central location for weighing and dosing. Approximately 50 % of the cattle were in the 151–200 kg BW class (49 %; 95 % credibility intervals [47%, 50 %]), smaller cattle (0–50, 51–100 and 101–150 kg BW) comprised 47 %, and heavier weights classes comprised only 4 %.

### Entomological impact

#### Mosquito identification

Overall, 1410 female mosquitoes were identified morphologically. The members of the *An. gambiae* s.l. and *An. funestus* s.l. complexes were further analysed by PCR. From the molecular analysis, 286 *An. gambiae* s.s., 173 *An. arabiensis* and 186 *An. funestus* s.s. were identified. The clay pots were not useful for the collection of *Anopheles* mosquitoes; only one *An. arabiensis* was collected by this method while the majority of the specimens were *Culex* spp.

#### Mosquito blood feeding patterns

**Fig. 1:**
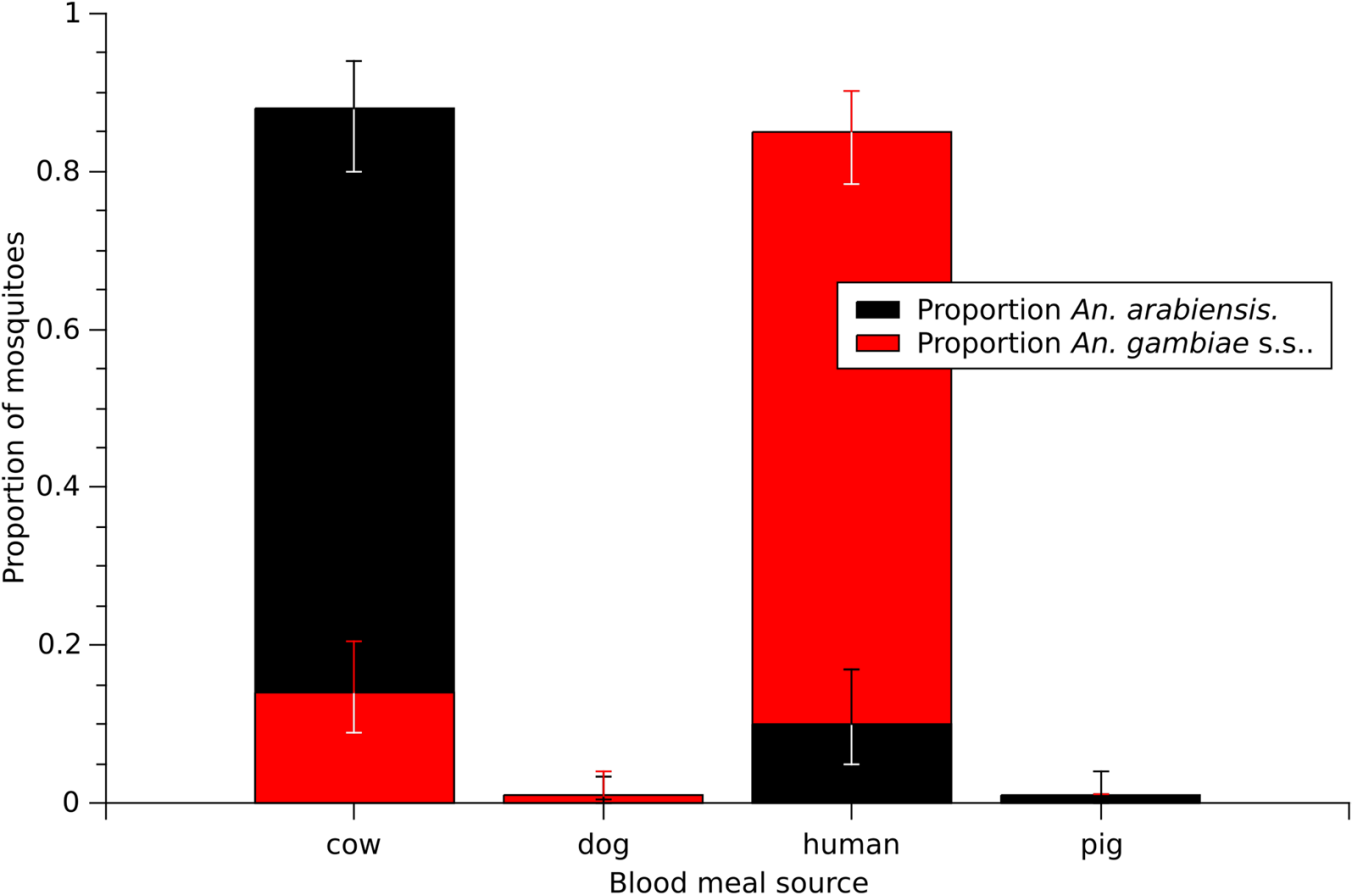
Distribution of *An. arabiensis* blood meals (n = 91 blood feeding events) and *An. gambiae s.s.* blood meals (n = 131 blood feeding events)

In total, 89/173 (51 %) *An. arabiensis* contained blood meals that were identifiable, 127/286 (44 %) *An. gambiae* s.s. had identifiable blood meals, and 55/186 (30 %) *An. funestus* s.s. had discernible blood meals. Blood from two different vertebrate hosts was detected in both *An. gambiae* s.s. (4/127) and *An. arabiensis* (2/89). In *An. arabiensis*, these mixed blood meals were cow + dog and human + cow. In *An. gambiae* s.s., all four mixed blood meals were human + cow. These mixed blood meals were treated as separate blood feeding events, for a total of 131 blood feeding events for *An. gambiae* s.s. and 91 blood feeding events for *An. arabiensis*. For *An. arabiensis*, 81/91 (89 %) of blood meals came from cattle, and 7/91 (8 %) were from humans (Fig. 1). The remaining identifiable blood meals were from dogs (2/91, 2 %) and a pig (1/91, 1 %). In contrast, 120/141 (85 %) of the discernible blood meals from *An. gambiae* s.s. were from human, 20/141 (14 %) were from cattle, and 1/141 (<1 %) blood meals from dogs (Fig. 1). All (55/55) blood meals from *An. funestus* s.s. were of human origin. Analyzing the blood meal proportions over the duration of the study no change was observed before and after treatment for our target species *An. arabiensis* (Table 1).

**Table 1:** *Anopheles arabiensis* blood meals from cattle and humans

	Baseline		1 WPT		2 WPT	
	N	Proportion	N	Proportion	N	Proportion
T cattle	13	0.76 [0.51, 0.93]	19	0.93 [0.77, 0.99]	7	0.94 [0.64, 1.00]
T human	5	0.20 [0.06, 0.45]	0	0.00 [0.00, 0.08]	0	0.01 [0.00, 0.21]
C cattle	9	0.86 [0.59, 0.98]	19	0.81 [0.63, 0.93]	11	0.96 [0.75, 1.00]
C human	1	0.09 [0.01, 0.35]	3	0.13 [0.03, 0.29]	0	0.00 [0.00, 0.14]

Values in square brackets represent the 95 % credibility intervals. Blood meals from dog and pig sources are not presented in the table. Mixed blood meals were added to their corresponding class

*T* treatment area, *C* control area

#### Sporozoite rates

**Fig. 2:**
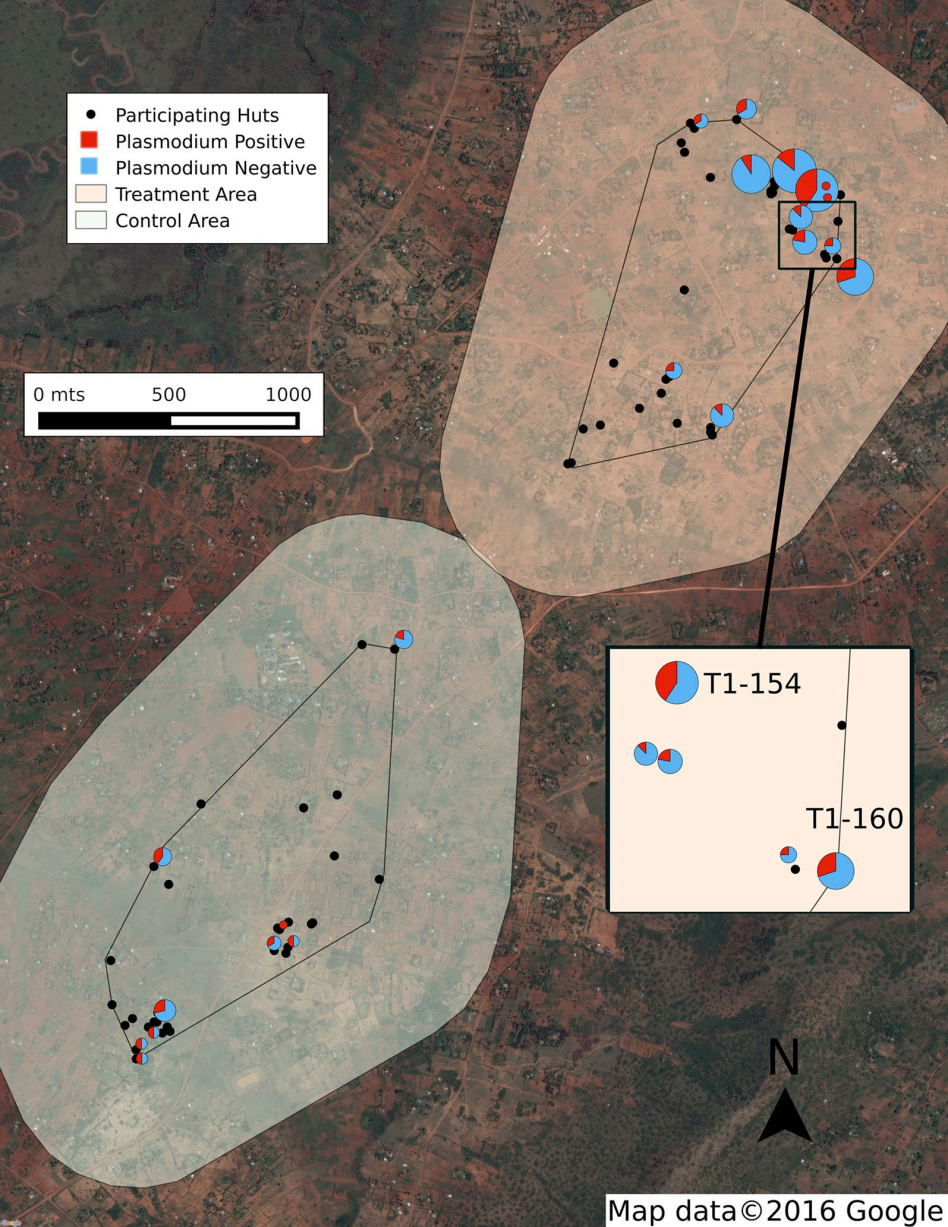
Distribution of sporozoite positive huts in the study area. *Pie charts* represent buildings with *P. falciparum* positive mosquitoes; *black dots* represent no positive mosquitoes found. The size of the *pie chart* is proportional to the number of collected mosquitoes

*Anopheles gambiae* s.s. had a significantly higher sporozoite rate than either *An. arabiensis* or *An. funestus* s.s. (probability 95 %; Table 2). The rate remained the same for the three species across control and treatment areas. Further analysis of the sporozoite rate for the *An. arabiensis* and *An. funestus* s.s. is not possible given the small number of *P. falciparum* positive mosquitoes found for these species. The high sporozoite rate from the treatment area in *An. gambiae* s.s. is mainly the result of two huts (T1-154 and T1-160; Fig. 2). These two huts, separated by 250 meters, contained 17 sporozoite-positive *An. gambiae* s.s.

**Table 2:** *Plasmodium falciparum* sporozoite rates

	*An. arabiensis*	*An. gambiae* s.s.	*An. funestus* s.s.
Positive/total T	1/74	34/202	2/90
Positive/total C	1/90	11/77	3/95
Sporozoite rate T	0.02 [0.001, 0.06]	0.17 [0.12, 0.22]	0.03 [0.004, 0.07]
Sporozoite rate C	0.02 [0.0002, 0.05]	0.15 [0.074, 0.23]	0.04 [0.007, 0.08]

The values in square parenthesis represent the 95 % credibility intervals

*Pos* positive, *Ctrl* control area, *Tre* treatment area, *Spo* sporozoite

Because of this extraordinarily high sporozoite rate for *An. gambiae* s.s., sporozoite-positive (=head/thorax positive for *P. falciparum*) mosquitoes collected from the same huts were analyzed with the hypothesis that the presence of individuals with febrile cases of malaria sleeping in those buildings provides some explanation for clusters of infected mosquitoes. Abdomen DNAs from the multiple sporozoite-positive specimens from the same hut, as well as sporozoite-negative mosquitoes from those huts, were screened for *P. falciparum*. Through this additional testing, mosquitoes which were sporozoite-positive (=head/thorax positive; abdomen—negative) were confirmed, and also identified mosquitoes for which only the abdomen was infected and contained human blood, and mosquitoes for which the head/thorax and abdomen were both positive. There were also multiple mosquitoes from these huts that were negative for *P. falciparum*. These results are suggestive that gametocyte-positive individuals sleeping in a few huts were serving as the source of infection for a disproportionately large number of mosquitoes.

#### Mosquito density

**Fig. 3:**
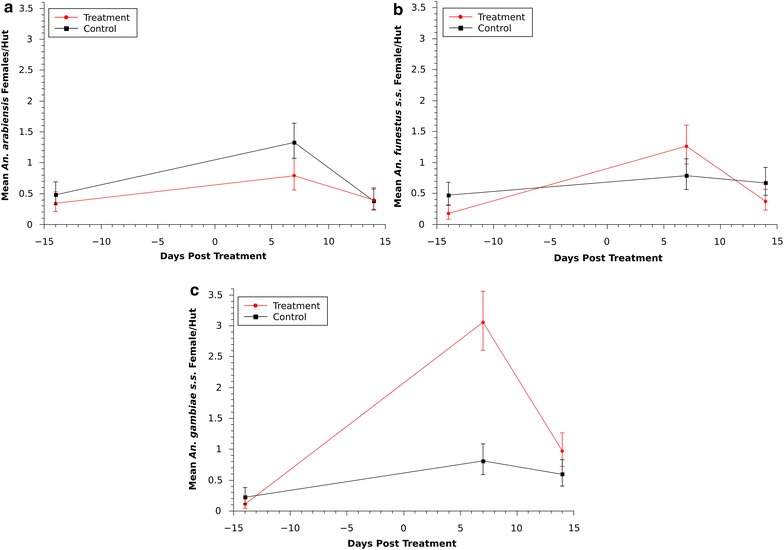
Indoor female resting density for **a** *An. arabiensis,* **b** *An. funestus* s.s. and **c** *An. gambiae* s.s. *Error bars* represent 95 % credibility intervals around the mean

During the study the most abundant species was *An. gambiae* s.s. (Fig. 3c) while the other two species were found in smaller indoor resting densities (Fig. 3a, b). All three species showed a significant increased between the baseline and one-week post-treatment (WPT) collections, which is especially noticeable for *An. gambiae* s.s. in the treatment site (Fig. 3c). All three species peaked, at one WPT, in both areas (control and treatment) suggesting the effect of environmental influences on the mosquito populations. However, while *An. gambiae* s.s. and *An. funestus* s.s. showed higher indoor resting densities in the treatment than the control area, the indoor resting density of *An. arabiensis* was significantly lower in the treatment area than in the control area (95 % probability; Control mean = 1.33 95 % CI [1.08, 1.64], Treatment = 0.79 [0.56, 1.07]). This difference demonstrates a significant reduction in indoor resting density of 38 % [(Control – Treatment)/Control × 100]. In the treatment area by the second week post-treatment the density of all three species was significantly lower when compared to the previous week. During the same period, the densities were also not significantly different across the control or treatment area in the three species. Overall, *An. gambiae* s.s. and *An. funestus* s.s. showed similar patterns (Fig. 3b, c respectively), but *An. gambiae* s.s. was found at a higher density.

## Discussion

Eprinomectin has long been used in the animal health industry for controlling endoparasites in cattle [34], but its utility as a public health tool to control ectoparasites has not been extensively investigated. While known to alter mosquito survivorship in the laboratory for up to 7 days significantly [35], eprinomectin had not been evaluated in the field for malaria vector control until now. In this study, treatment of cattle with eprinomectin had an immediate effect on the indoor resting density of *An. arabiensis*. This vector population reduction of 38 % was observed for approximately one week in accordance to what was shown in cattle shed experiments [35]. Treating humans with ivermectin, another macrocyclic lactone in the avermectin family, Alout et al. [18] also found a significant effect on mosquito survivorship for approximately one week. In that study, a 33.9 % reduction in survivorship of *An. gambiae* s.s. was observed for seven days following mass drug administration of ivermectin in humans. While this effect was also brief, a significant reduction in mosquito parity rates was observed for more than two weeks following treatment, and sporozoite rates were reduced by 77.5 % for 15 days [18]. While the current study did not follow the extended entomological impact of eprinomectin on mosquito infection rates or parity, measuring these parameters in future field studies is important.

Although the indoor resting densities of *An. arabiensis* were reduced by 38 % for one week, an increase in *An. gambiae* s.s. and *An. funestus* s.s. was observed (Fig. 3). Populations of all three species increased during that first-week post-treatment, presumably due to some external factors, but it is not clear why indoor resting densities of *An. gambiae* s.s. and *An. funestus* s.s. were significantly greater in the treatment area for only that period during which a decrease in *An. arabiensis* was observed. Further investigation is necessary to study the interspecies dynamics and competition for resting sites and determine if a reduction in the population of *An. arabiensis* in the treatment area could be related to a corresponding increase of *An. gambiae* s.s. collected indoors. If this is the case, an integrated approach may be necessary by using treatment of cattle in combination with ITNs, IRS, human prophylactics, or a combination to counteract any increase in exposure to *An. gambiae* s.s. while *An. arabiensis* populations are reduced. Bayoh et al. [4] documented a decrease of *An. gambiae* s.s. over multiple years in western Kenya due to the use of ITNs and a resulting proportional increase of *An. arabiensis*. Therefore, following the long-term changes in relative abundance of these species as a result of various malaria control interventions is paramount.

In the current study clay pots were not useful to collect *An. arabiensis*. Similarly, sampling small numbers of *An. arabiensis* has been reported for Kenya by Mutuku et al. [36]. Sampling with clay plots was stopped by Mutuku et al. [36] because *An. gambiae* s.l. was recovered in small numbers in spite an extensive sampling effort. The modifications to the Odiere et al. [21] methodology most likely had a negligible impact because all pots were inspected visually after the first shake and if insects were still present the process was repeated. The lack of samples from outdoor resting places do not detract from this study results because there is no evidence that indoor resting *An. arabiensis* females constitute a sub-population [37]. Resting place is most likely a result of convenience for the female mosquito while host preference is driven by genetics [37].

No change was observed in the proportion of blood meals as a result of treatment in our target species. For the duration of the experiment *An. arabiensis* significantly preferred cattle over humans as expected since host preference is primarily a genetic trait [37, 38], though it can be modified by the abundance of hosts. In this study the number of cattle and people was similar between the control and treatment area and a change in host preference would most be unlikely given the study time frame.

This study also identified malaria infection “hot spots” within the study area, in which a disproportionately large number of infected mosquitoes were collected from few huts (Fig. 2). The “20/80 rule” has been described for some infectious agents including *Plasmodium*, in which a particular core group of individuals, representing 20 % of the population, contribute at least 80 % to the transmission potential of a pathogen [39, 40]. In these huts, mosquitoes were actively becoming infected at the time of collection. Sporozoite-negative mosquitoes containing infected human blood meals were collected alongside many infected mosquitoes, already sporozoite-positive. Interestingly, these huts with many sporozoite positive mosquitoes also had the highest numbers of resting mosquitoes in general (Fig. 2), suggesting some mechanism of attraction to the house with a sick person. By removing the mosquitoes from the two huts with the most sporozoite positive mosquitoes from the analysis, the SR sporozoite rate would be reduced from 0.15 to 0.11.

Spatial heterogeneity of malaria infections has been described previously in Kenya and elsewhere at multiple spatial scales [41–43], although from a clinical perspective. Bejon et al. [41] presented data from demographic surveillance linked to passive case detection in Pingilikani dispensary in Kilifi District, coastal Kenya. Data were collected from 1500 homesteads within an 8 km radius followed for nine years. Their analysis identified hot spots within hot spots, with one significant hotspot (p = 0.016) comprised of a single homestead, in which there were 36 episodes of malaria [41]. In Tanzania, Mosha et al. [42] also not only identified spatial clusters of human infections, but demonstrated the relative stability of hotspots, in that clusters of infection and seropositivity were predictive of future disease in those same houses. The relative stability of hot spots has been linked to whether the nature of clinical surveillance performed, with hotspots of asymptomatic parasitemia being more stable over time than hotspots of febrile malaria [41, 44]. This phenomenon has also been demonstrated in the highlands of western Kenyan [45]. Zhou et al. [45] found that while hotspots of asymptomatic infections remained unchanged over time, new clusters of clinical malaria cases emerged in the uphill areas during the peak season. The method of case surveillance being used and whether febrile or asymptomatic cases are being monitored was, therefore, important in identifying hotspots and predicting their relative persistence over time. The uniqueness of the presented data is that hot spots at the level of homestead were identified through mosquito collections rather than patient screening. Whether the mosquitoes were becoming infected from asymptomatic carriers or febrile cases is unknown. Further studies should investigate the stability and seasonality of these hot spots, and how these data might be incorporated into a malaria surveillance and elimination programme.

## Conclusions

Malaria transmission in the Samia District of western Kenya is driven by *An. gambiae* s.s., *An. funestus* s.s. and *An. arabiensis*. While the former two species are highly anthropophilic, *An. arabiensis* fed predominantly on cattle in the study area. When cattle were treated with the systemic drug eprinomectin for two consecutive weeks, the indoor resting density of *An. arabiensis* was decreased by 38 % in the treatment area for at least seven days. The long-term entomological and epidemiological impact of this population reduction, as well as the effect of a more sustained treatment regimen, have yet to be determined. Future studies should also follow up on the interspecies dynamics between *An. gambiae* s.s. and *An. arabiensis* in response to vector-control interventions targeting one or the other species, as well as the focal nature of infectious (gametocyte-positive) patients serving as super-spreaders to the local mosquito population. Eprinomectin treatment of cattle has the potential to impact significantly residual, outdoor malaria transmission driven by *An. arabiensis*, and may be a valuable tool to supplement existing vector control interventions which target the most anthropophilic species.
